# Meetings of the mind!

**DOI:** 10.1007/s13187-016-1071-9

**Published:** 2016-06-29

**Authors:** Arthur Michalek

**Affiliations:** University at Buffalo, Buffalo, NY USA

In a few short months, there will be a gathering of the clan to celebrate the 50th anniversary of the first annual meeting of the American Association for Cancer Education (AACE). I would encourage all who read this editorial to make plans to attend. In addition to the regular fare of presentations on innovative cancer education programming, there will be special presentations commemorating our 50th. The AACE, and the annual meeting, has undergone numerous changes since it was formed, but has remained true to its mission “…of bringing together members and invited guests for discussion and demonstrations related to cancer teaching activities and varying pedagogical views…” [1]. In an earlier editorial [2], I recounted the history of our Association which harkens back to the 1940s when it was formed as a resource for grantees of the National Cancer Institute’s Undergraduate Cancer Training Grants. Meetings of this group continued through 1966 when at the 19th meeting the Group formally organized as the AACE. The annual meeting continues to serve as a resource of innovation and an avenue of communication among like minded professionals, but has become more expansive and representative in its membership. Attendance at these meetings now includes members of the Cancer Patient Education Network (CPEN) as well as our colleagues from the European Association for Cancer Education (EACE). This expansion has most recently resulted in a change of name to the annual meeting to the “International Cancer Education Conference.” It is the largest meeting that focuses solely on the state of cancer education. This year’s meeting will take place 14–16 September in Bethesda, Maryland (visit aaceonline.com for more info). The theme for the meeting is “Promoting Cancer Education, Equity, and Precision Medicine Globally.” Dr. Maria Bishop and her Program Committee are to be commended for assembling what appears to be a superb meeting. The main sessions will include a host of invited speakers including Dr. Douglas Lowy, Acting Director of the National Cancer Institute (NCI); Dr. Edward Trimble the Director of the Center for Global Health at the NCI; Dr. Julia Rowland the Director of Cancer Survivorship (NCI); Dr. Ming Lei, Deputy Director of the Cancer Training Branch (NCI); Dr. Jeannette Korczk, Program Director of the Cancer Training Branch (NCI); Dr. Elmer Huerta, Director of the Cancer Preventorium at Washington Cancer Institute; and Dr. Olivia Carter-Pokras, Interin Associate Dean of Diversity and Inclusion at the University of Maryland School of Public Health. This group will bring a wealth of experience to our meeting and their insights invaluable to our development of relevant, effective programs. I personally cannot wait to hear what they have to say and trust that you are equally curious.

The annual meeting will also offer a number of workshops where attendees can gain new knowledge or hone their skills. As of this writing, confirmed workshops (and their leaders) include: Cancer Survivorship (Noreen Aziz MD, PhD, MPH); Genetics and Cancer Care (Kathleen Blazer, EdD, MS, Cathy Calzone, MSN, PhD, and Jeffrey Weitzel, MD); Health Literacy (Cathy Meade, PhD, RN, FAAN); Palliative Care (Elaine Wittenberg, PhD); and Use of a Translator (Maria Bishop, MD). An expansive collection of workshops that will surely meet the needs of every attendee. As a past participant and presenter at our workshops, I can attest to their value. Dialog begun in these workshops continues through the year and is a positive influence on one’s research and/or program development.

The annual meeting proper will include both invited speakers as well as concurrent sessions. Sessions will cover the entire cancer education waterfront from highly competitive abstracts submitted for presentation. Another beneficial aspect of the meeting is the Poster Sessions where we get an opportunity to personally meet and query presenters from around the world. These are always such great learning and networking opportunities. In commemoration of our 50th anniversary, there will be a luncheon panel of the History of Cancer Education. One of the highlights of the annual meeting is the presentation of the Samuel Harvey lecture which will be given this year by Dr. Huerta. We will also hear from Dr. Charles Kelly the President of the EACE on Shared Decision Making as an Educational Tool in Cancer Management. I trust that this preview of the annual meeting has whet your appetite. Please visit the website to register for what will undoubtedly be a dynamic meeting.

I would like to reemphasize that the meeting is now known as the “International Cancer Education Conference.” This title more accurately reflects the diversity and applicability of topics and presenters at the meeting. No one individual exemplifies this more than Dr. Amr Soliman (current President) who is internationally known for his efforts in cancer education and cancer epidemiology. He, as well as a number of our members, have given lectures and developed training programs throughout North Africa, the Middle East, and Europe. I have had the honor of traveling with him and witnessing his dedication and proficiencies. I have also benefited from attending meetings of the EACE. The 2015 meeting in Heidelberg, Germany, was particularly insightful as I came to meet Radislaw Tarkowski, MD, PhD and a group of his students from Wroclaw Medical University in Poland. I was very impressed by the number and quality of their presentations such that I had numerous conversations with Dr. Tarkowski at the meetings and continued these upon my return to the States. Our conversations began to focus on the development of a 2-week training program at Wroclaw University focused on the art of scientific publishing and the principles and methods of cancer education research. A noble goal, but where to find funding? Our answer resided with the Fulbright Scholar Program. This program awards grants to US faculty/professionals to engage in short-term collaborative projects. Many of you have likely heard of the Fulbright which provides funding to support long-term exchanges, but they also operate a Fulbright Specialist Program (cies.org/program/Fulbright-specialist-program) which provides funding for shorter periods (2 to 4 weeks). This allows, as they state, “greater flexibility to pursue projects that work best with their current academic or professional commitments.” This is a two step process requiring the application/review/approval of the Specialist and a separate application/review/approval of the host institution. We found the application process to be quite straight forward and administered by knowledgeable, responsive professionals. I would recommend that anyone interested review their website and consider applying. This program is named for Senator William J. Fulbright (Arkansas) who was a gifted statesman who saw the benefit of scholarly exchanges as a means of advancing mutual understanding. An impactful program named after a visionary Senator. The Specialist Program allowed us to develop a 2-week program for medical students and even faculty.

Wroclaw is the fourth largest city in Poland (population approximately 650,000). Wroclaw Medical is one of the most renowned public medical schools in Poland and offers a 6-year curriculum taught in English. The school curriculum, like so many others, focuses on clinical care and does not offer sufficient time for medical research let alone those areas aligned with cancer education. The program that Dr. Tarkowski and I developed incorporated didactic lectures, workshops, and private/group consultations. Full details of this experience will be presented in a future manuscript in this Journal that Dr. Tarkowski and I will co-author. I found the attendees to be highly motivated, intelligent, and engaged. While we both had initial concerns over how active the students would be, these reservations were laid to rest early in the first session. The students were highly inquisitive and asked highly focused questions which often lead to an ever expansive and detailed conversation. I cannot praise the students enough for their level of participation. Many brought completed or manuscripts in progress for discussion at the workshops. These discussion not only lead to a strengthening of their manuscripts, but the discussions addressed areas of interest to all participants which should lead them to ever better study design. I also cannot thank Dr. Tarkowski enough for his leadership and for his gracious generosity as a host. I came to know the land of my ancestors at a very personal level. My wife (Terry O) and I not only became more keenly aware of the rich history of Polonia but also benefited from its rich culinary experience. I encourage all to explore the many benefits of the Fulbright Program.

Be well and Na zdrowie!

AMM
